# Supplementary material for: The ribosome modulates folding inside the ribosomal exit tunnel
Source: Commun Biol. 2021 May 5;4:523. doi: 10.1038/s42003-021-02055-8 (PMC8100117; doi:10.1038/s42003-021-02055-8)
Supplement: Supplementary file 1 — Supplementary Information [file 42003_2021_2055_MOESM1_ESM.pdf]

Supplementary Figures for

The ribosome modulates folding inside the ribosomal exit tunnel

Florian Wruck[1], Pengfei Tian[2], Renuka Kudva[3], Robert B. Best[4], Gunnar von Heijne[3,5], Sander J. Tans[1, *] and Alexandros Katranidis[6, *]

[1] AMOLF, Amsterdam, The Netherlands

[2] Protein Engineering, Novozymes A/S, Kongens Lyngby, Denmark.

[3] Department of Biochemistry and Biophysics, Stockholm University, Stockholm, Sweden

[4] Laboratory of Chemical Physics, National Institute of Diabetes and Digestive and Kidney Diseases, National Institutes of Health (NIH), Bethesda, MD, USA

[5] Science for Life Laboratory, Stockholm University, Solna, Sweden

[6] Institute of Biological Information Processing IBI-6, Forschungszentrum Jülich (FZJ), Jülich, Germany

[*] For correspondence: a.katranidis@fz-juelich.de and tans@amolf.nl

C-terminal linker 26aa - **ADR1a inside the exit tunnel**

ATG TAG CAT CAC CAT CAC CAT CAC TCT AAA CAA ATC GAA GTA AAC CGGG TCT
AAA CCG TAT CCG TGC GGC CTG TGC AAC CGC TGC TTT ACC CGC CGC GAT CTG
CTG ATT CGC CAT GCG CAG AAA ATT CAT AGC GGC AAC TAG GGT GGA GGT TCT
GGT GGA GGT TCC TTC AGC ACG CCC GTC TGG ATA TGG TGG TGG CCT CGC ATC
CGT GGC CCC CCT

C-terminal linker 34aa - **ADR1a outside the exit tunnel**

ATG TAG CAT CAC CAT CAC CAT CAC TCT AAA CAA ATC GAA GTA AAC CGGG TCT
AAA CCG TAT CCG TGC GGC CTG TGC AAC CGC TGC TTT ACC CGC CGC GAT CTG
CTG ATT CGC CAT GCG CAG AAA ATT CAT AGC GGC AAC TAG GGT GGT GGT TCT
GGT GGT GGA TCT GGT GGA GGT TCT GGT GGA GGT TCC TTC AGC ACG CCC GTC
TGG ATA TGG TGG TGG CCT CGC ATC CGT GGC CCC CCT

Supplementary Fig. S1. Nucleotide sequence of ADR1a constructs. Sequence of the two constructs used in this study. The sequence of ADR1a is highlighted green and the four amino acids that bind the Zn^{2+} ion are underlined. The amber and the 4-base codons, used for the incorporation of unnatural amino acids are highlighted red. The 6xHis tag is highlighted blue and the SecMstr arrest peptide orange. The two constructs differ only in the length of the C-terminal linker upstream of the arrest peptide.

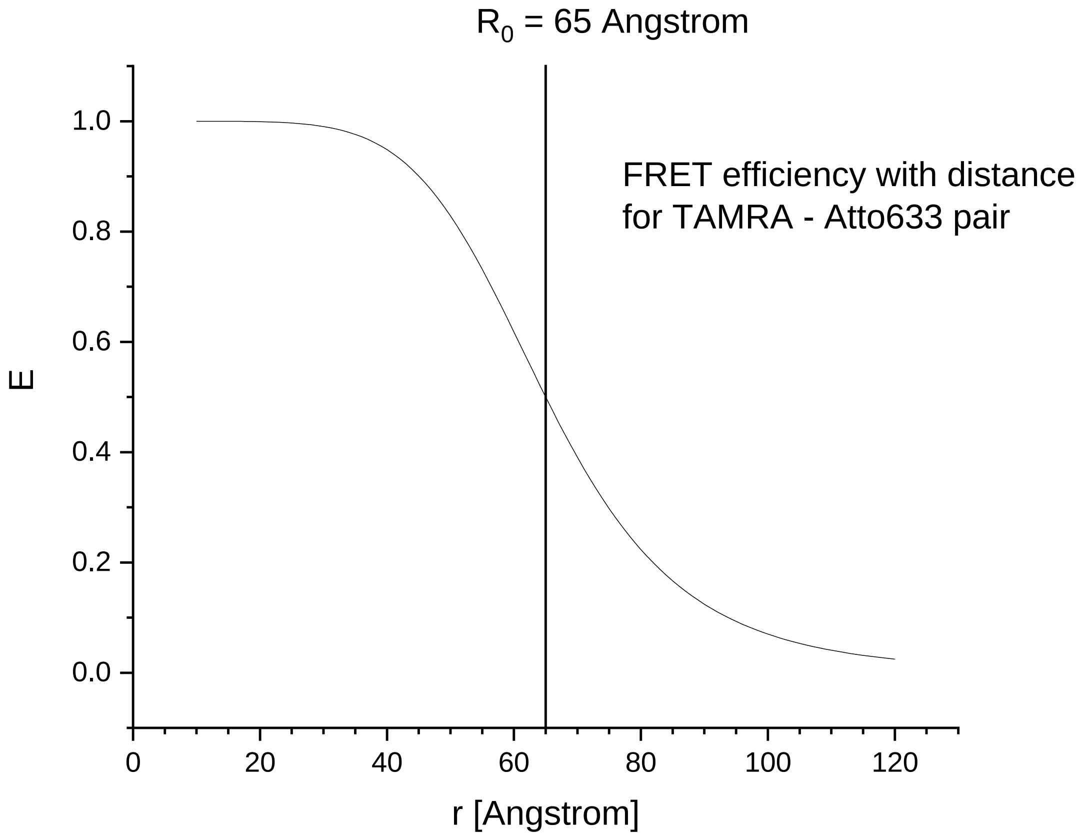

Supplementary Fig. S2. FRET efficiency for TAMRA – Atto633 dye pair. The change in energy transfer as a function of distance between the two dyes with a value of R_0 = 65 Å.

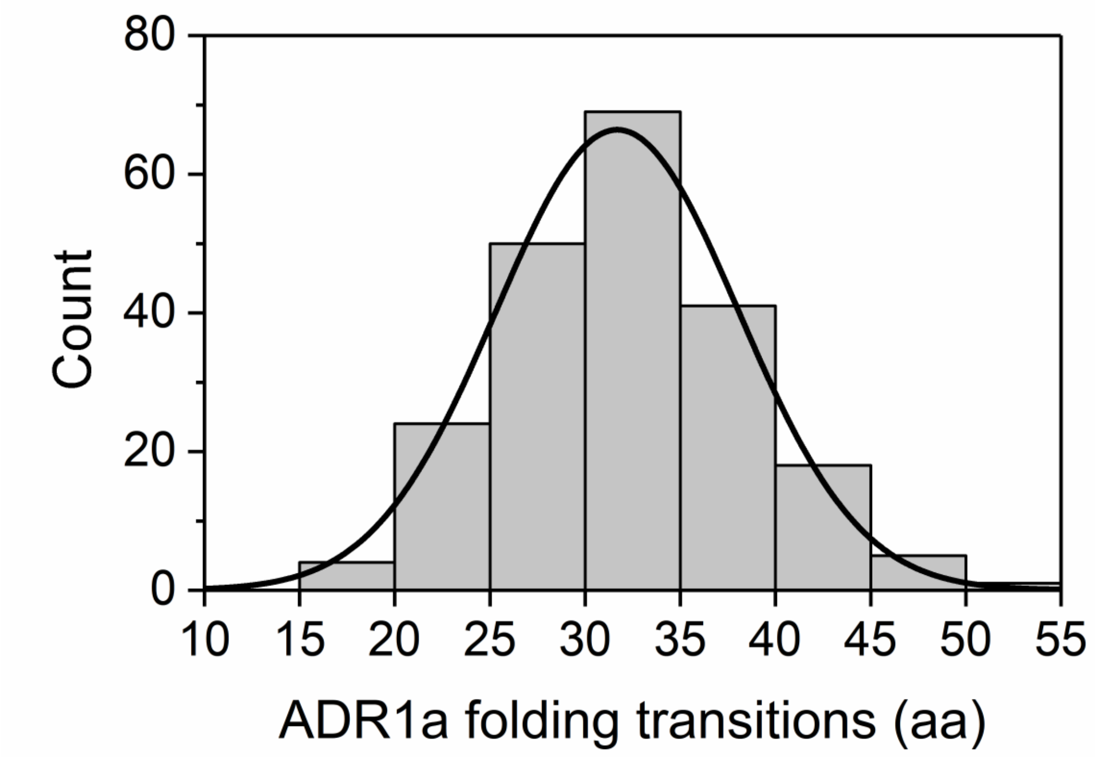

Supplementary Fig. S3. Histogram of ADR1a unfolding and refolding transitions measured with the optical tweezers. The number of amino acids unfolding (refolding) during a transition. The transition length is measured in nm and converted to amino acids using the Worm-Like Chain (WLC) model, as shown before (8).

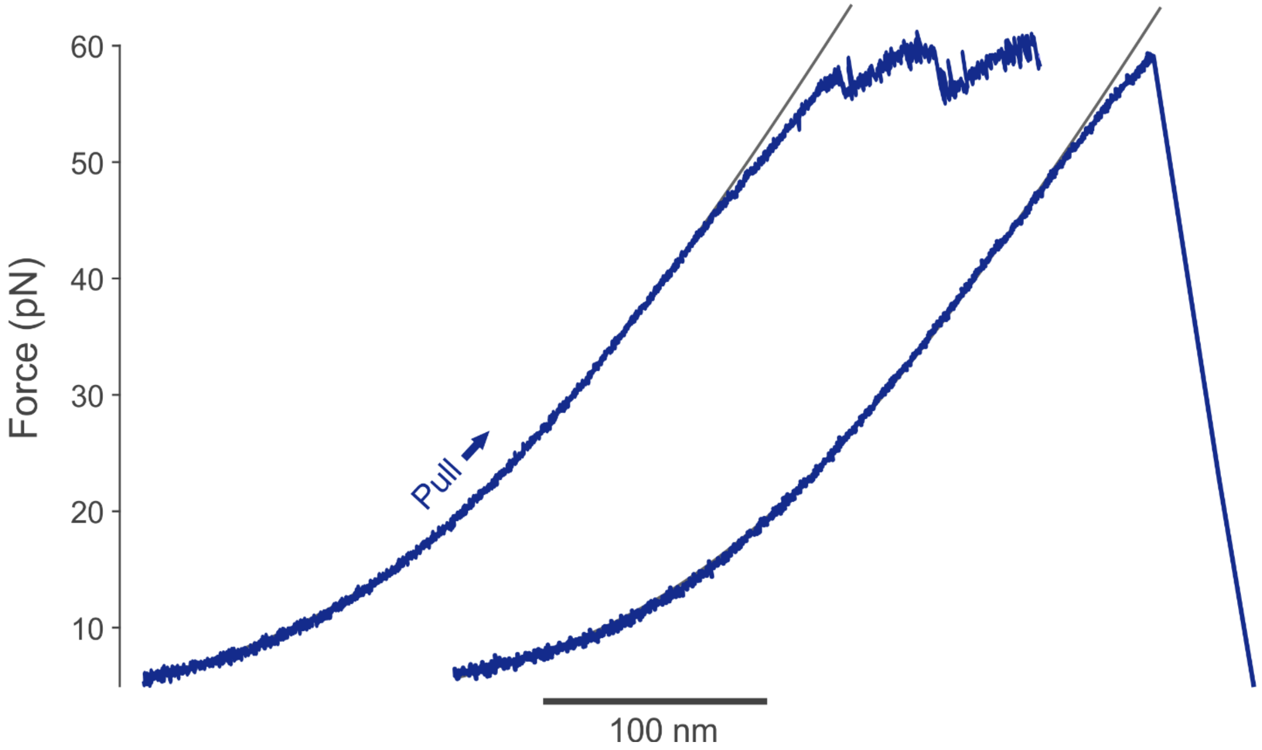

Supplementary Fig. S4. Control experiment on nascent chains lacking ADR1a. Force-extension traces of optical tweezers experiments on a nascent peptide that was all-linker (pRSET + amber + 2x Gly/Ser + SecMstr), where a repeat of the original Gly/Ser linker of the ADR1a construct L= 34 was added to ensure that the construct spans the entire length of the ribosomal tunnel so that the N-terminal biotin could still be tethered in situ. A deviation of the curve from the eWLC model above 35 + pN, partly due to twisting and stretching of the rotationally unconstrained DNA handles, can be seen. The final force decrease indicates rupture of the tether. These data are consistent with a lack of the ADR1a protein chain segment, as ADR1a-like unfolding was observed (n = 24 molecules).

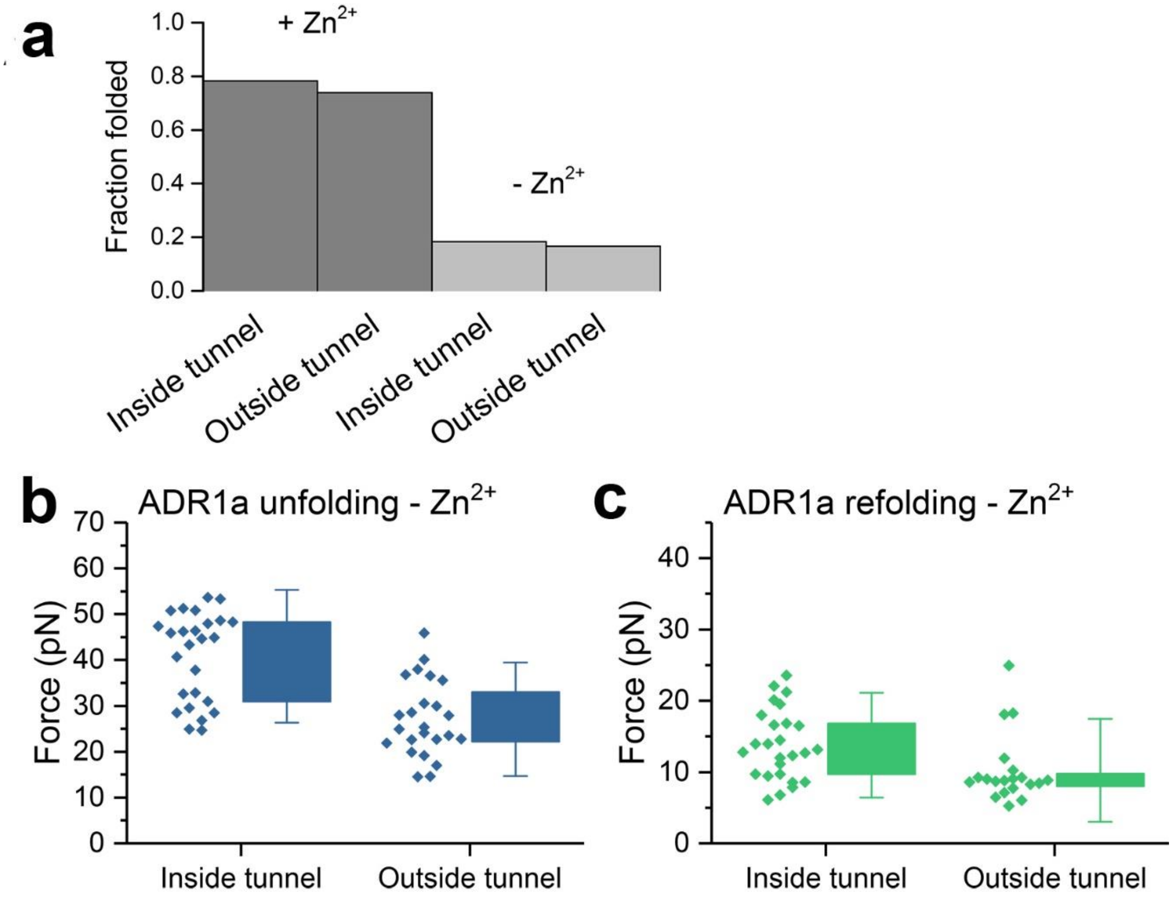

Supplementary Fig. S5. Unfolding and refolding of ADR1a with the optical tweezers in the absence of Zn^{2+}. (a) Fraction of pulling cycles that showed refolding of ADR1a. In the absence of Zn^{2+} (50 µM TPEN) ADR1a refolds for 18.3 % of all pulling cycles (n = 142 molecules) inside the ribosomal tunnel and 16.7 % of pulling cycles (n = 144 molecules) outside the tunnel. In contrast, in the presence of 50 µM Zn^{2+} ADR1a refolding was observed for 78.3 % of all pulling cycles (n = 175 molecules) inside and 73.9 % of all pulling cycles (n = 119 molecules) outside the ribosomal tunnel. **(b)** Unfolding and **(c)** Folding force distributions of ADR1a inside and outside of the ribosomal tunnel in the absence of Zn^{2+} (50 µM TPEN). The box plots show the interquartile range of the data between the 25th and 75th percentiles, the whiskers represent the standard deviation; the individual data points are plotted alongside.

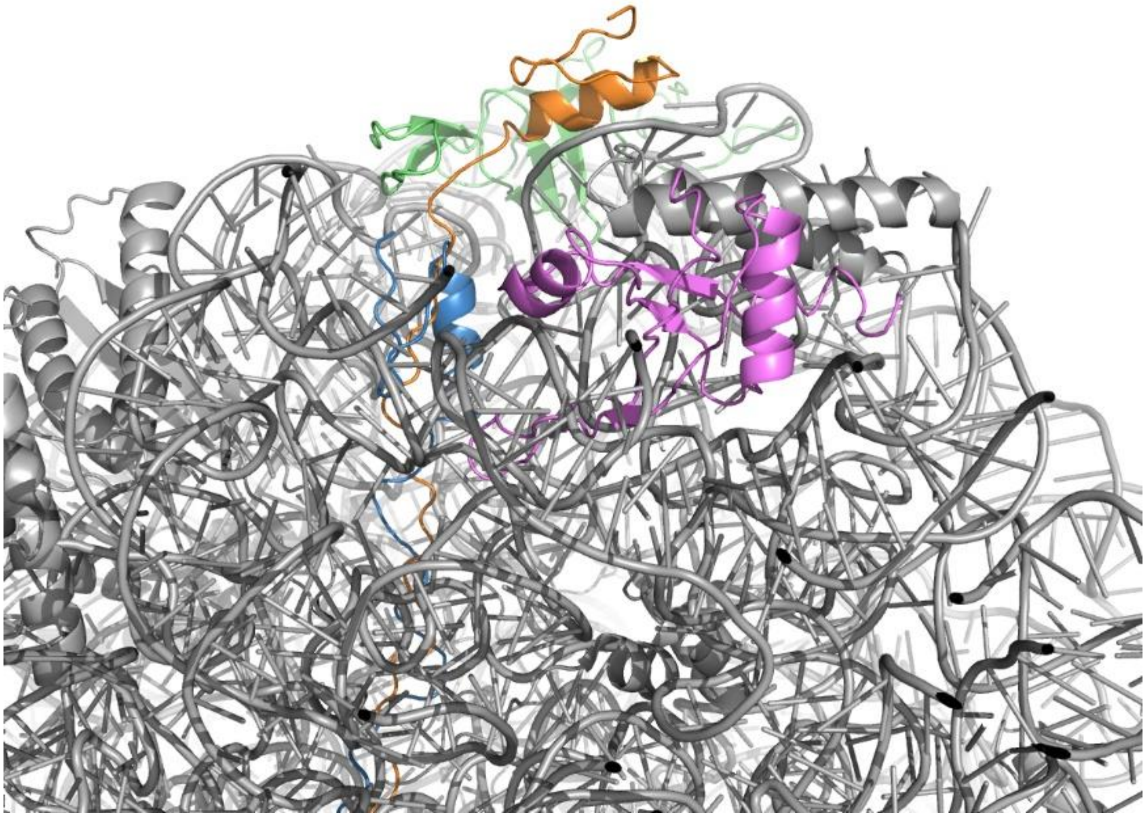

Supplementary Fig. S6. MD snapshot of ADR1a and ribosome. Folded ADR1a under force (F=10 pN) on the ribosome. ADR1a with L=26 inside the ribosomal tunnel is colored in blue and with L=34 at the mouth of the tunnel in orange. Ribosomal proteins L23 and L24 are colored in green and purple, respectively.

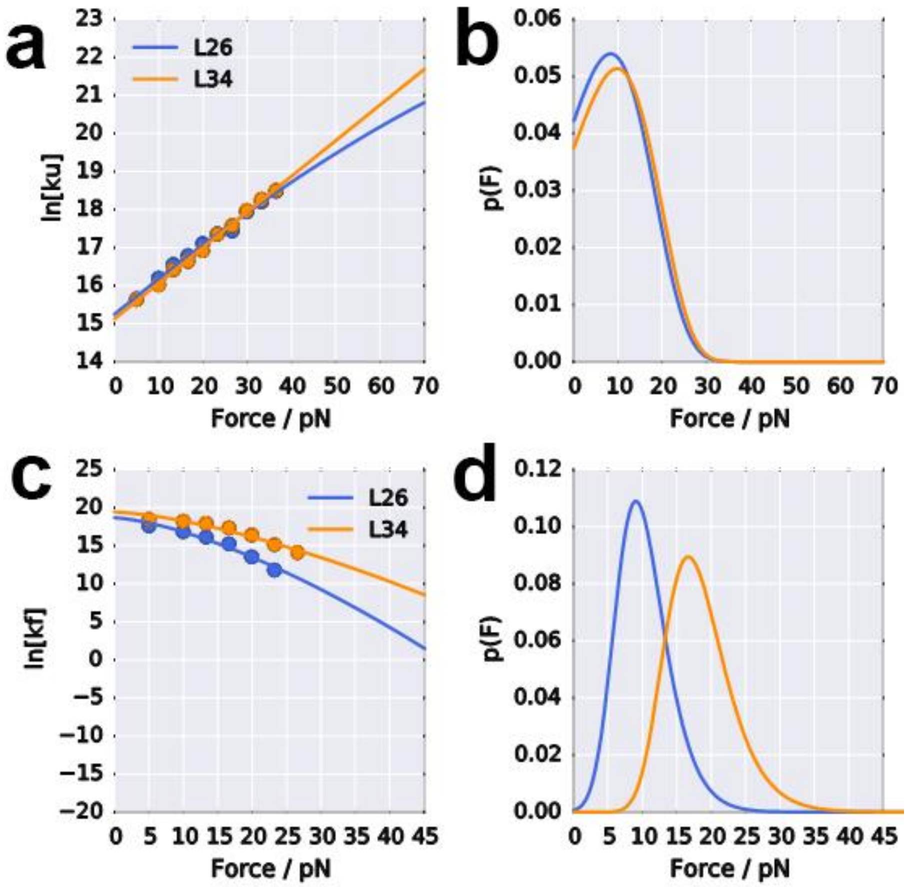

Supplementary Fig. S7. Repulsive model. In this model no electrostatic interactions are taken into account between ADR1a and the ribosome. **(a)** Simulated rates of ADR1a unfolding under different pulling forces. **(b)** Corresponding probability distribution of the simulated force required to unfold the ADR1a. **(c)** Simulated rates of ADR1a folding on the ribosome under different pulling forces. **(d)** Corresponding probability distribution of the simulated force required to refold the ADR1a.

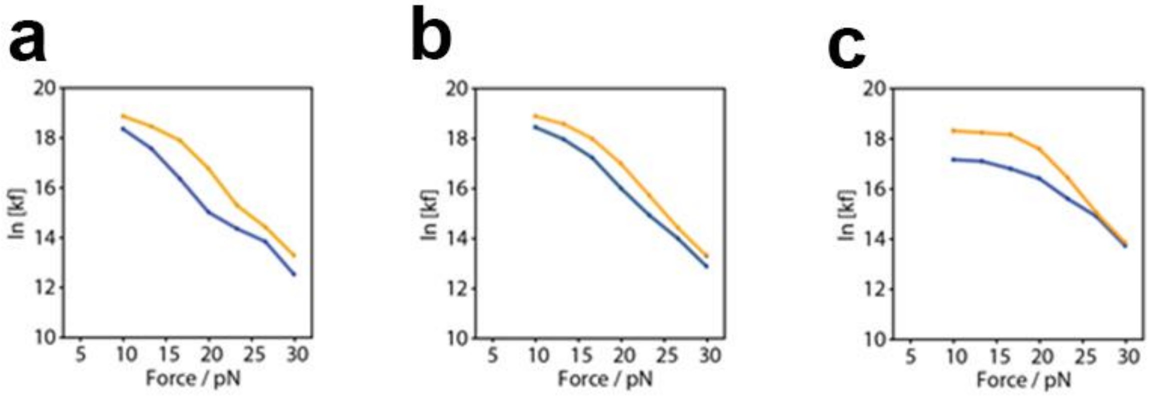

Supplementary Fig. S8. Model with non-specific interactions. Force dependence of the folding rates for RNC-ADR1a (L=26, blue) and RNC-ADR1a (L=34, orange) including attractive interactions between the nascent chain and ribosome from MD simulations. **(a)**, **(b)** and **(c)** are with different attractive strengths between the nascent chain and ribosome (ε). ε=0.1, 0.3 and 0.6 kJ mol^{-1} respectively.
