# Supplementary material for: The ribosome modulates folding inside the ribosomal exit tunnel
Source: Commun Biol. 2021 May 5;4:523. doi: 10.1038/s42003-021-02055-8 (PMC8100117; doi:10.1038/s42003-021-02055-8)
Supplement: Supplementary file 9 — Reporting Summary [file 42003_2021_2055_MOESM9_ESM.pdf]

nature research

Corresponding author(s): Dr. Alexandros Katranidis

Last updated by author(s): Feb 27, 2021

Reporting Summary

Nature Research wishes to improve the reproducibility of the work that we publish. This form provides structure for consistency and transparency in reporting. For further information on Nature Research policies, see our Editorial Policies and the Editorial Policy Checklist.

Statistics

For all statistical analyses, confirm that the following items are present in the figure legend, table legend, main text, or Methods section.

n/a	Confirmed	
☐	☒	The exact sample size (*n*) for each experimental group/condition, given as a discrete number and unit of measurement
☐	☒	A statement on whether measurements were taken from distinct samples or whether the same sample was measured repeatedly
☐	☒	The statistical test(s) used AND whether they are one- or two-sided<br>*Only common tests should be described solely by name; describe more complex techniques in the Methods section.*
☐	☒	A description of all covariates tested
☐	☒	A description of any assumptions or corrections, such as tests of normality and adjustment for multiple comparisons
☐	☒	A full description of the statistical parameters including central tendency (e.g. means) or other basic estimates (e.g. regression coefficient) AND variation (e.g. standard deviation) or associated estimates of uncertainty (e.g. confidence intervals)
☒	☐	For null hypothesis testing, the test statistic (e.g. *F*, *t*, *r*) with confidence intervals, effect sizes, degrees of freedom and *P* value noted<br>*Give P values as exact values whenever suitable.*
☒	☐	For Bayesian analysis, information on the choice of priors and Markov chain Monte Carlo settings
☒	☐	For hierarchical and complex designs, identification of the appropriate level for tests and full reporting of outcomes
☒	☐	Estimates of effect sizes (e.g. Cohen's *d*, Pearson's *r*), indicating how they were calculated

Our web collection on statistics for biologists contains articles on many of the points above.

Software and code

Policy information about availability of computer code

Data collection	Tweez-O-Matic version 36.0, running under LabView 11.0 was used to acquire force-displacement data with the C-trap optical tweezers provided by Lumicks. Fluorescence kymographs were acquired with Lumicks software Scanary 3.4
Data analysis	All analysis was performed using custom made scripts in Matlab R2016b and python 3.6, available upon request

For manuscripts utilizing custom algorithms or software that are central to the research but not yet described in published literature, software must be made available to editors and reviewers. We strongly encourage code deposition in a community repository (e.g. GitHub). See the Nature Research guidelines for submitting code & software for further information.

Data

Policy information about availability of data

All manuscripts must include a data availability statement. This statement should provide the following information, where applicable:

- Accession codes, unique identifiers, or web links for publicly available datasets
- A list of figures that have associated raw data
- A description of any restrictions on data availability

Data that support findings of this study are available from the corresponding author upon reasonable request. Original data to produce plots shown in Figs. 3, 4 and 5 are also available upon request.

Field-specific reporting

Please select the one below that is the best fit for your research. If you are not sure, read the appropriate sections before making your selection.

☒ Life sciences ☐ Behavioural & social sciences ☐ Ecological, evolutionary & environmental sciences

For a reference copy of the document with all sections, see nature.com/documents/nr-reporting-summary-flat.pdf

Life sciences study design

All studies must disclose on these points even when the disclosure is negative.

Sample size	No statistical methods were used to predetermine sample size. Sample sizes were chosen based on previous experience and published studies to assess reproducibility. Experiments were repeated multiple times on multiple substrate molecules, which were sufficient to obtain the described statistical significance.
Data exclusions	Only data from measurements of single tethers between the optically trapped beads, that could be fitted with the worm-like chain (WLC) model, were included. Multiple tethers (no DNA overstretching plateau at 65 pN) and tethers featuring damaged DNA handle characteristics (following ssDNA WLC model) were excluded.
Replication	All experiments were replicated multiple times, using different bead pairs and substrate molecules. All attempts at replication were successful.
Randomization	Samples were not randomized in the experiments. Randomization was not applicable as samples were allocated according to different conditions such as longer or shorter stalled nascent chain length.
Blinding	Experiments were not blinded as the data acquisition and analysis were done in different conditions

Reporting for specific materials, systems and methods

We require information from authors about some types of materials, experimental systems and methods used in many studies. Here, indicate whether each material, system or method listed is relevant to your study. If you are not sure if a list item applies to your research, read the appropriate section before selecting a response.

Materials & experimental systems

n/a	Involved in the study
☐	☒ Antibodies
☒	☐ Eukaryotic cell lines
☒	☐ Palaeontology and archaeology
☒	☐ Animals and other organisms
☒	☐ Human research participants
☒	☐ Clinical data
☒	☐ Dual use research of concern

Methods

n/a	Involved in the study
☒	☐ ChIP-seq
☒	☐ Flow cytometry
☒	☐ MRI-based neuroimaging

Antibodies

Antibodies used	Roche Diagnostics Germany, Anti-digoxigenin bodies, Cat. No. 11333089001, polyclonal antibody from sheep
Validation	The polyclonal antibody from sheep is specific to digoxigenin and digoxin and shows no cross-reactivity with other steroids, such as human estrogens and androgens.
