# Supplementary material for: The ribosome modulates folding inside the ribosomal exit tunnel
Source: Commun Biol. 2021 May 5;4:523. doi: 10.1038/s42003-021-02055-8 (PMC8100117; doi:10.1038/s42003-021-02055-8)
Supplement: Supplementary file 11 — Description of Additional Supplementary Files [file 42003_2021_2055_MOESM11_ESM.pdf]

## Description of Additional Supplementary Files

**File name:** Supplementary Data 1

**Description:** Source data of Figs. 2a and 2b.

**File name:** Supplementary Data 2

**Description:** Source data of Figs. 3a and 3b.

**File name:** Supplementary Data 3

**Description:** Source data of Supplementary Figs. S5b and S5c.

**File name:** Supplementary Data 4

**Description:** Source data of Fig. 4.

**File name:** Supplementary Data 5

**Description**: Source data of Supplementary Fig. S7.

**File name:** Supplementary Data 6

**Description:** Source data of Supplementary Fig. S8.

**File name:** Supplementary Data 7

**Description:** Source data of Figs. 5a and 5b.
